# Job Crafting: Older Workers’ Mechanism for Maintaining Person-Job Fit

**DOI:** 10.3389/fpsyg.2017.01548

**Published:** 2017-09-08

**Authors:** Carol M. Wong, Lois E. Tetrick

**Affiliations:** Department of Psychology, George Mason University, Fairfax VA, United States

**Keywords:** job crafting, older workers, person-job fit, proactivity, successful aging

## Abstract

Aging at work is a dynamic process. As individuals age, their motives, abilities and values change as suggested by life-span development theories (Lang and Carstensen, 2002; Kanfer and Ackerman, 2004). Their growth and extrinsic motives weaken while intrinsic motives increase (Kooij et al., 2011), which may result in workers investing their resources in different areas accordingly. However, there is significant individual variability in aging trajectories (Hedge et al., 2006). In addition, the changing nature of work, the evolving job demands, as well as the available opportunities at work may no longer be suitable for older workers, increasing the likelihood of person-job misfit. The potential misfit may, in turn, impact how older workers perceive themselves on the job, which leads to conflicting work identities. With the traditional job redesign approach being a top-down process, it is often difficult for organizations to take individual needs and skills into consideration and tailor jobs for every employee (Berg et al., 2010). Therefore, job crafting, being an individualized process initiated by employees themselves, can be a particularly valuable mechanism for older workers to realign and enhance their demands-abilities and needs-supplies fit. Through job crafting, employees can exert personal agency and make changes to the task, social and cognitive aspects of their jobs with the goal of improving their work experience (Wrzesniewski and Dutton, 2001). Building on the Life Span Theory of Control (Heckhausen and Schulz, 1995), we posit that job crafting, particularly cognitive crafting, will be of increasing value as employees age. Through reframing how they think of their job and choosing to emphasize job features that are personally meaningful, older workers can optimize their resources to proactively redesign their jobs and maintain congruent, positive work identities.

## Introduction

The concept of fit has always been a topic that receives attention from both researchers and practitioners in the field of organizational psychology, as person-environment fit has significant implications for employees’ attitudes and behaviors, as well as organizational outcomes (Edwards, 1991; Kristof, 1996). Person-environment fit can be broadly defined as the compatibility resulting from the characteristics of an employee and his/her work environment being well aligned (Kristof-Brown et al., 2005). As there are various unique components of the work environment, breaking down the construct of environment helps elucidate how congruence with each distinct part could impact employees’ attitudes and behaviors. Specifically, this paper focuses on person-job fit, which refers to the degree of alignment between the individual and the job he/she holds (Edwards, 1991). Person-job fit can be achieved in two ways: when employees’ skills match the specific job requirements (abilities-demands fit), and when their needs are congruent with the opportunities available at work (needs-supplies fit). Similar to other levels of fit, person-job fit is related to positive attitudes, job performance and personal well-being (Park et al., 2011; Afsar et al., 2015). Furthermore, early research focused on static fit, examining aspects of the person and the environment that are stable over time. However, it is currently recognized that achieving fit is a dynamic process, with features of both employees and jobs changing over time (Tinsley, 2000). Changes in the work environment can result in changes in employees (e.g., restructuring production process corresponds to new training for factory workers), and changes in employees can also lead to changes in the work environment (e.g., advancement of employees’ technological knowledge can further transform the production process). This notion of dynamic fit is useful in considering the effects of aging in the organizational context. Given that there are increasing proportions of older workers in the active workforce, it is important to examine the within-individual age-related changes as well as changes in jobs on employees (Feldman and Vogel, 2009).

This article aims to answer the question of how job crafting would be a useful strategy for workers to achieve greater fit as they move forward with their careers. In the first section, we explore the aging-related changes that employees may face. The aging process contributes to major changes of different forms, including gains, maintenance and decline, in almost all aspects of individuals’ capabilities and interest, such as cognitive skills, physical abilities, emotions and motives. As people age, growth and extrinsic motives weaken while intrinsic motives increase (Kooij et al., 2011), which may result in employees prioritizing different crafting strategies (i.e., task crafting, relational crafting, and cognitive crafting). Despite common misconceptions about aging, these changes vary considerably across individuals. General statements that describe the average trends of aging in workers may not adequately describe how changes would impact individuals and how they perform on their jobs. However, without proper modifications to job requirements, these within-person age-related changes can substantially alter both abilities-demands fit and needs-supplies fit, improving or worsening the overall person-job fit. In the second section, we discuss how changes on the job itself may also contribute to potential misfit. As the nature of the job evolves over time, altered job expectations placed upon employees may create challenges for workers and require them to reorient how they perform their job. The potential misfit may impact how older workers perceive themselves on the job, which leads to conflicting individual work identities. Building on the Life Span Theory of Control (Heckhausen and Schulz, 1995), we posit that job crafting, particularly cognitive crafting, will be of increasing value as workers age. Although cognitive crafting has received little research attention thus far, it can help individuals to cope with age-related changes and maintain favorable work identities. It also aids the development and maintenance of task and relational crafting by directing their resources to areas that are personally meaningful. The increasingly limited resources due to aging make the selection of suitable job crafting practices to be of greater importance for older workers. Unlike the standardized initiatives implemented by organizations, the individualized, bottom-up approach of job crafting would be able to accommodate the substantial individual differences among older employees. Thus, a combination of different forms of job crafting allows them to realign their abilities and needs with their job to develop and maintain congruent work identities.

## Who are Older Workers?

Depending on the purpose and field of study, older workers have been defined using a cut-off age varying from 40 to 75 years old (Stein and Rocco, 2001). While the age of 40 is frequently used based on the United States Age Discrimination in Employment Act of 1967 (ADEA), organization decision markers identify older workers as those who reached the age of 52 years (McCarthy et al., 2014). Some researchers also have treated age as a continuous variable and not adopted a specific age distinguishing older workers from younger workers (e.g., Ng and Feldman, 2010). Clearly, there is no consensus in the literature regarding the definition of older workers.

While we understand the argument of using a specific age to denote older workers can be significant in workplace practices, multiple perspectives need to be taken into account to understand this unique population. Because aging is a continuous process, its effects on workers do not begin or stop at a particular point. Using a specific cut-off does not capture a complete picture of how the multidimensional, age-related changes may affect employees’ crafting activities. In addition, there is considerable variability in the lifespan trajectories, depending on individual and contextual characteristics as well as their interaction (Baltes, 1987). Even among employees at the end of the age distribution, there can still be multiple subgroups such as “early retirees,” the “young-old,” and the “old-old” (Pinquart, 2001). Using a single age to divide the workforce into groups is overly simplistic. Moreover, the definition is constantly shifting in response to contextual factors. The increased average life expectancies allow more older adults to participate in the workforce, exemplified by the fact that the largest segment of the world’s working population is 45–49 years old (Ng and Feldman, 2010). The demographics of particular industries can also influence who are considered to be older workers. Therefore, this paper employs the lifespan developmental perspective to examine workers’ job crafting in response to the physiological, psychological and social changes resulting from aging as well as the time-related changes in the job.

## Age-Related Changes in Person

### Physical Changes

Normal aging involves a multitude of physiological changes, primarily in sensory function, muscle function, cardiovascular function and immune response (Maertens et al., 2012). Individuals may experience reduced visual acuity and hearing sensitivity, decline in aerobic capacities (which leads to decrease in heart rates and increase in blood pressure), as well as reduced psychomotor speed and abilities with the increase in age (Forteza and Prieto, 1994). There is also a robust literature that documents the loss of physical strength associated with aging, which occurs due to bone loss, as well as the decline in both muscle tone and muscle mass (Warr, 1994). However, it is important to note that these changes are gradual, and that vast individual differences impact older workers’ job experience in various ways, especially since white-collar jobs now constitute an increasingly large proportion of the labor force. For example, the age-related decline in physical strength, endurance and speed may hinder performance of those whose jobs rely heavily on physical abilities. As a result, an older grocery clerk may no longer be able to stock shelves due to the heavy lifting involved. Instead, he/she may job craft to assume only the responsibilities of a cashier or incorporate the usage of transport dollies to complete the stocking tasks. However, the potential impairments in performance due to aging would not be consistent among all individuals, even if they hold the same job position.

Aging is also related to decline in homeostasis, the reduced ability for the body to maintain and return to normal operations across different situations (Hedge et al., 2006). Older workers may have less tolerance for extreme physical job conditions such as heat and cold due to their reduced abilities to withstand temperature changes. It is also more difficult for older workers to adjust to non-standard shift work, as it would take them longer to recover from altered sleep patterns (Blok and de Looze, 2011). This can be a particular challenge for older employees with jobs that require constant adjustment to different work conditions. For instance, construction work which requires shift schedules and high physical demands can become increasingly difficult as workers age.

Surveys have found a higher percentage of adults between the age of 45 to 64 reporting their health as good or excellent compared to that from 25 years ago, and rates of functional limitations have also decreased (James et al., 2013). In some sense, older workers have become healthier. However, despite no increase in subjective physical health problems, meta-analytic evidence suggests that employees still experience modest decline in physical health such as elevated blood pressure, cholesterol and insomnia as they get older (Ng and Feldman, 2013). The reduced efficiency of the immune system also makes older workers more prone to illnesses, and require longer recovery time. Studies have proposed that the length of sick leave is positively correlated with age (Thomson et al., 2000). Older workers also recover more slowly from injuries (Sterns et al., 1985). Therefore, although the incidence of injuries is lower for older workers (Ng and Feldman, 2008), jobs with high risk for injuries may become increasingly unsuitable as workers get older.

In response to these changes in physical abilities, employees may be prompted to employ different forms of job crafting, such as using cognitive crafting to make sense of these changes, and alter how they perceive and perform their job tasks accordingly.

### Cognitive Changes

Cognitive abilities are another functional area that shows age-related changes, which include both gains and losses. There is a negative relationship between age and fluid intelligence (Gf), such as processing speed, working memory, and selective attention (Truxillo et al., 2015). It is exemplified by the Seattle Longitudinal Study (Schaie and Hertzog, 1983; Schaie, 1994), which followed several cohorts from diverse backgrounds over their life courses. Results suggested that all abilities (with the exception of perceptual speed which starts to decrease in the early 1930s) begin to decline in the mid 1940s. However, crystallized intelligence (Gc), which is the accumulated knowledge, skills, and wisdom, continues to grow until late life. The gains in crystallized intelligence can allow older workers to compensate for losses in fluid intelligence (Warr, 2001). This may help explain the lack of a significant negative relationship between age and job performance in the literature (Ng and Feldman, 2013), since most jobs nowadays require a combination of both types of intelligence. Older workers may also counteract declining cognitive abilities by performing their jobs more conscientiously (Farr and Ringseis, 2002) and increasing their effort at work (Bunce and Sisa, 2002). Age-related cognitive changes may lead to person-job misfit for those without the ability to redesign their jobs through job crafting.

### Skills

According to Fossum et al. (1986), deterioration in present skills or the failure to acquire new ones as job requirements change result in obsolescence of employees’ skills. Older workers’ skills may deteriorate over their work histories. In addition, they are often stereotyped as less adaptable, having lower ability to learn and being more difficult to train (Ng and Feldman, 2008; Posthuma and Campion, 2008). Companies, therefore, are reluctant to invest in their training, under the assumption of lower return on investment due to the perceived lack of potential for development associated with age. Organizational practices often discourage older employees from engaging in training (Maurer, 2001; Farr and Ringseis, 2002). In addition, when older workers have access to training, it is often of shorter duration and lower quality (Felstead et al., 2010). In reality, older workers value opportunities to maintain their capabilities for a better sense of job security (Herrbach et al., 2009). It is also a driving factor for individuals to return to work post-retirement (Armstrong-Stassen, 2008), suggesting that developmental options need to accommodate the needs of older workers in order to be motivating. The lack of access to suitable opportunities may harm their self-efficacy and impact how they perceive themselves at work (Maurer and Tarulli, 1996). They may face a higher risk of their skills becoming obsolete, which interferes with their abilities to perform effectively on the job and thus contributes to poorer person-job fit. Older workers may, for example, job craft by attending training workshops on their own initiative to update their skills in response.

### Emotions

Emotional regulation is a functional area in which individual gains are observed as the aging process unfolds (Scheibe and Zacher, 2013). Older individuals have learned to effectively regulate their emotions and developed emotional resiliency in stressful situations through their work histories and other life experiences (Carstensen and Mikels, 2005). The change in future time perspective from Socioemotional Selectivity theory (which will be discussed below), in which older adults tend to perceive time as a more limited resource, also helps explain the age-related differences in affective responses. Instead of attending to the negative aspects of the environment, individuals tend to shift their focus to the positive cues which provide immediate emotional gratification (Mather and Carstensen, 2005), in part due to their sense of limited time remaining. Therefore, older workers are more likely to utilize positive emotions in the face of work-related problems (Folkman et al., 1987), which could lead them to job craft differently compared to their younger colleagues. When vertical career advancement is no longer feasible, older workers may shift their focus to broadening their job tasks and utilizing other strategies to make their job more enjoyable. Or, they may cognitively reframe to perceive an increase in job demands as a challenge, which may prompt them to engage in other crafting behaviors such as seeking support from colleagues.

## Motives

The major lifespan development theories and empirical literature support that individuals’ motives change over time. Therefore, the value of certain activities and outcomes, such as close colleagues, promotion opportunities, and pay, change accordingly. These theories inform our understanding of the dynamic nature of work motives and how these changes can contribute to person-job misfit and the job crafting strategies for older workers.

### Intrinsic Work Motives

The stereotype that older workers are less motivated has been shown to be inconsistent with the cumulated research evidence (Ng and Feldman, 2012), but it is important to differentiate what exactly motivates older workers. Intrinsic work motives refer to integral parts of the work that satisfy individuals’ psychological needs. Some examples of intrinsic work motives include needs for autonomy, achievement and social connection with others. There is a common notion that general growth motives weaken during aging. In other words, older workers are less concerned with general learning compared to their younger counterparts, which aligns with what is suggested in developmental theories (Baltes, 1997; Kanfer and Ackerman, 2004). A meta-analysis of 15 empirical studies including a total sample size of over 6,000 employees found a weak, negative relationship between age and learning motivation (Ng and Feldman, 2012). The Selection, Optimization and Compensation (SOC) theory indicates that limited personal resources (such as time and cognitive capacity) become more strained as individuals become older (Baltes, 1997). The allocation of resources then shifts from growth to maintaining the resources already possessed, keeping losses to a minimum. As a result, SOC theory predicts a negative relationship between age and growth-related motives. However, it is important to distinguish how different types of growth motives change with age. Although there is ample evidence corroborating that knowledge acquisition motives decline with age, meta-analytic evidence indicates a positive relationship between age, need for autonomy and achievement (Kooij et al., 2011). As general life expectancy has increased, it is not uncommon for individuals to live into their 1980s and 1990s. Yet, the age range for individuals remaining in the active workforce is narrower. It is likely that the significant decline in intrinsic growth motives proposed would not be realized until after retirement. Given the significant individual differences in aging, it is likely that some older employees still value training opportunities in order to maintain their competencies and satisfy their need for achievement.

As individuals grow older, their values regarding social interactions may undergo changes. Socioemotional Selectivity theory (SST; Carstensen, 1995; Carstensen et al., 1999) introduced future time perspective (FTP) – when individuals view time as expansive, they prioritize goals that aim at optimizing the future. Thus, younger workers, who tend to have expansive FTP, are inclined to pursue opportunities that are useful in the more distant future, such as acquiring additional work-related knowledge (Kooij and Van De Voorde, 2011). When people perceive time is running out with increasing age, they may gravitate toward goals that are emotionally fulfilling. The constrained FTP steers the focus to the utilization of skills and social interactions that affirm positive self-concept and promote instant emotional satisfaction. In the context of work, older workers are thought to prefer deepening existing core relationships, such as those with close colleagues who share similar interests, over broadening peripheral relationships. Kanfer and Ackerman (2004) similarly proposed that generativity motives and the importance of protecting one’s work self-concept increase with age. Compared to advancing their careers, older workers are more likely to focus on passing knowledge onto their younger colleagues (Mor-Barak, 1995; Kooij and Van De Voorde, 2011). However, empirical data provides equivocal results on this link. Several studies have found a negative relationship between age and need for affiliation with others (Fagenson, 1992; Mudrack and Naughton, 2001); however, meta-analytic evidence based on 35 empirical studies including over 29,000 employees suggests that age is unrelated to social motives at work (Kooij et al., 2011). One possible explanation for the variability is that the need for social affiliation is met in domains outside of work, which may alter how older workers craft their work relationships.

### Extrinsic Work Motives

Extrinsic work motives are job features and outcomes that occur as a consequence of work, such as compensation, social recognition and benefits. Extrinsic growth motives generally refer to the valence placed on promotions and advancement at work. As suggested by Kanfer and Ackerman (2004), the salience of these extrinsic outcomes decrease with age. The shift in temporal perspective proposed by SST and meta-analytic data (Kooij et al., 2011) provide evidence supporting this change. Older workers may not believe that they can realize their future-oriented opportunities. The idea of limited time remaining in their careers drives older workers to focus on fewer but specific outcomes that would provide immediate gratification, such as a sense of achievement from accomplishing challenging job tasks. In addition, most studies have found no relationship between age and need for recognition (Churchill et al., 1979; Inceoglu et al., 2009), which also aligns with the SOC theory. As individuals grow older, they also gain better understanding about their strengths and have clearer professional identities (Helson et al., 1995). In fact, most individuals become more confident and emotionally stable over the life span (Roberts et al., 2006). As a result, praise and need for recognition from others may not have much of an impact on how older workers’ perceive themselves. These age-related changes do suggest that as employees age they will be motivated to engage in job crafting to align the outcomes of work with their personal motives.

## Time-Related Changes in Jobs

Other than changes within individuals, elements of work change over time as well. While changes in job responsibilities and within occupations may alter workers’ job demands, older workers may also achieve more job autonomy as they advance their careers. In addition, adoption of technology and Human Resources Management (HRM) policies at work have the potential to improve or impair their person-job fit.

### Job Demands

As employees get older and advance through the various career stages, their jobs may become more demanding due to the increased level of responsibility associated with longer tenure (Hurrell and Lindström, 1992). For example, mid-career employees face higher job demands compared to those earlier in their careers, as they assume more supervisory duties but have yet to achieve full job autonomy (Ng and Feldman, 2010). Other research has suggested that job demands decrease as employees move toward retirement, because they either get assigned less challenging responsibilities or have voluntarily modified their employment terms to part-time (Feldman and Ng, 2007). Prior studies proposed that jobs can be stereotyped into young-typed and old-typed, based on the age of prototypical job incumbents (Perry and Finkelstein, 1999). While young-typed jobs have been associated with tasks that rely on technology and ability to adapt quickly, old-typed jobs are those that require extensive organization-specific knowledge or experience (Kaufman and Spilerman, 1982; Perry, 1994). Similarly, prototype matching can be extended to the assignment of job tasks: older workers may not be considered to be a good fit for tasks that are commonly associated with younger workers. For example, despite being technologically literate and having the same qualifications, an older worker may be less likely to be considered for a challenging project working with a technology company. In addition, older workers face more negative perceptions of their job performance from their supervisors (Hassell and Perrewe, 1995). Based on these age stereotypes, older workers may receive narrower sets of tasks, which could shape their work experience. Older workers, who are often in the maintenance career stage, are concerned with preserving interest in their jobs and getting involved in areas that appeal to them (Conway, 2004). The lack of challenges at work may result in older workers perceiving themselves as less competent compared to their younger colleagues and finding their jobs not as intrinsically motivating. This may influence how older workers engage in job crafting.

### Changes within Occupations

Changes in the routines and activities within an occupation occur over time, which would also lead to cascading effects on job demands and, in turn, influencing person-job fit (Feldman and Vogel, 2009). For example, billing and insurance processes have become progressively more complicated in healthcare, while the amount of litigation for the industry has also increased. As a result, jobs of healthcare professionals have evolved to reflect these changes, as they face additional demands working with insurance carriers and malpractice lawyers (Feldman, 2013). Apart from the healthcare industry, multiple waves of a nationally representative surveys of teachers indicate an increase in work hours since the implementation of No Child Left Behind (Grissom et al., 2014). The performance-based legislation also alters teachers’ professional practices, resulting in modification of curriculum to reflect state standards and incorporate test-taking skills (Barrett, 2009). Both are examples of how changes within occupations over time would ultimately reshape job duties and potentially increase job demands. As older workers tend to have longer tenure and reduced career mobility, they are more likely to stay within the same occupations and jobs. Without suitable support in terms of resources, higher job demands and altered responsibilities may contribute to person-job misfit over time, which may prompt different forms of job crafting.

### Technology

Adoption of new technology can be a double-edge sword for older workers’ person-job fit. Technology facilitates the automation of work tasks, which in turn increases knowledge job demands (Drucker, 2000). This creates opportunities for older workers to shift away from physically arduous duties and diversify their job tasks, such as taking on new responsibilities that rely on their extensive domain knowledge. For instance, wearable safety glasses equipped with communication functions allow veteran building maintenance workers to offer their expertise in real-time to their younger colleagues, without having to climb to significant heights (Griffith, 2014). Telecommuting and blended work allow for time-independent and location-independent work, removing potential barriers that prevent older employees from continuing to work beyond retirement age (Dropkin et al., 2016). Older workers also show comparable performance on multiple telecommuting tasks to their younger colleagues (Sharit et al., 2004). They may proactively incorporate technology in their jobs accordingly to improve their overall work experience.

On the other hand, technology can also place higher demands upon older workers. When software is designed without accounting for age-related perceptual and cognitive changes, it may create additional challenges for older employees. Decline in color perception and visual acuity may make it particularly difficult for older workers to read obscure information on computer screens (Charness and Boot, 2009). As technological devices continue to shrink in size (Thompson and Atkins, 2010), more precise motor control is needed for proper usage. Reduced button size has been shown to result in increased time on tasks and higher mental workload for older users (Fezzani et al., 2010). In addition, given their age-related changes, effective technology-based training for older workers should be self-paced, highly structured, and incorporate a user-friendly and consistent interface to enhance learning outcomes (Williams van Rooij, 2012; Wolfson et al., 2014). These design issues in emerging workplace innovations create potential systematic barriers for older workers’ performance, and may hamper their confidence and motivation to fully utilize technology. In addition, information and communication technology has become an integral part of work, which can lead to unexpected strain. Interruptions from instant messaging and email alerts increase mental load, and such disturbances have been suggested to create more stress and lead to lower performance in older workers (Tams and Hill, 2017). While robots and other programs can perform an increasing variety of tasks to augment workers’ capabilities, workers also find them to be threatening to their job security (Cascio and Montealegre, 2016). As the impact may be particularly severe for older employees due to their lower job mobility, altering their perceptions through cognitive crafting can be one way to counteract such impact on their work experience.

The rapid pace of evolution in technology makes it difficult for workers, regardless of age, to stay abreast, which may result in gaps in their skills needed to perform well on the job. However, to the best of our knowledge, few studies have directly examined technology usage and its impact specifically for older workers. As attitude toward technology was strongly related to work motivation among older employees (Elias et al., 2012), individuals who struggle with incorporating technology in their work may have chosen to retire early since they cannot cope with such job demands. Therefore, those who are still active participants in the workforce may not face as many functional limitations in this area as previously theorized. In addition, because of the considerable differences in aging trajectories, more empirical data is needed to examine how technology in the workplace shapes older workers’ job crafting strategies, and in turn, their job experience.

### Human Resources Management (HRM)

The experience of HRM policies differs across employees due to both uneven implementation of practices and individual differences (Clinton and Guest, 2013). Organizations can be reluctant to invest in older workers since their younger counterparts would have more years remaining in their careers for companies to benefit from their investment (Schultz, 1961). Along with the stereotypes of lower potential for development, older workers have less access to training (Farr and Ringseis, 2002). As the perceived availability of training resources positively relates to employees’ self-efficacy (Maurer and Tarulli, 1996), older workers may attribute the lack of equal access to developmental opportunities as a sign of deficits in their own abilities, threatening their work identities. It is also common for stereotypes to influence important human resource decisions, such as training and performance evaluations. During subjective performance appraisal, age stereotypes may be invoked despite no conscious intent from the supervisors. Prior research has shown that older workers often receive lower performance scores even when they have the same qualifications as their younger counterparts (Posthuma et al., 2012). They may view themselves negatively because of the different evaluation criteria, which may prompt specific crafting practices in order to protect their identities and maintain self-esteem. As organizational practices can be seen as signals from employers to employees, adopting specific HR policies can be indicative to employees to cultivate a positive sense of self. For instance, distinct bundles of High-Performance Working Systems (HPWS), which are designed to enhance employees’ skills and empowerment, convey the idea that organizations value their employees. Research has found that the relationships between maintenance HR practices (e.g., performance appraisal) and well-being, as well as developmental HRM practices (e.g., training) and performance, strengthen with age (Kooij et al., 2013). In addition, HRM practices designed for older workers (such as formally recognizing their achievements and explicitly investing in their training) can counteract negative environmental cues of stereotype threat of ageism and reaffirm older workers’ social identity (Kulik et al., 2016). Older workers shifting their attention to cues set by companies through HRM policy implementation may help them sustain a positive sense of self.

## Job Crafting

Despite changes in abilities, needs and work motives being similar among older workers, there is still substantial between-individual variability in their aging trajectories. For instance, although future time perspective tends to become more limited, with a shorter time frame as people age (Carstensen et al., 1999), some older individuals may have more open-ended future time perspectives in regards to their careers. Since job redesign traditionally is a top-down process implemented by organizations, standardized policies are rarely able to take individual needs and abilities into consideration (Hackman and Oldham, 1976). Therefore, although one way to reduce the discrepancy between person and job fit would be job redesign, it is often difficult for organizations to update jobs for every single employee (Berg et al., 2010). This makes managing such a heterogeneous group of employees particularly challenging. In addition, older workers often face age stereotyping at work, which threatens their self-identity. Identity theory proposes that given a mismatch between identities and actions, people would proactively align their actions with expectations, as well as prioritize and integrate identities to cope with the discrepancy (Stryker and Burke, 2000). In fact, researchers have provided increasing evidence suggesting older workers exercise agency and adopt an active role in altering their perceptions, behaviors and environments at work to achieve fit (Freund and Baltes, 1998; Wahl et al., 2012). We propose that job crafting, being an individualized, bottom-up approach, provides a valuable avenue for older workers to adapt to their individual age-related changes, as well as the dynamic nature of work, to stay motivated in their job. Cognitive crafting, in particular, enables them to reframe their perceptions of the job and focus on job features that are personally meaningful, which further aids other forms of job crafting.

### Forms of Job Crafting

Job crafting is defined as changing the boundaries and conditions of job tasks, work relationships and the meaning of the job (Wrzesniewski and Dutton, 2001). While job design assumes employees are passive recipients of changes imposed by organizations, job crafting considers them to be active participants who restructure their own job boundaries as needed. It is a self-initiated behavior by employees with the goal to improve their own work experience. Unlike other proactive behaviors such as personal initiative and role innovation, job crafting does not necessarily lead to positive organizational outcomes, as the focus is on the employees themselves. Through engaging in job crafting, employees are able to exert personal control over their jobs, to establish positive self image, and to satisfy their need for fulfilling interpersonal relationships (Wrzesniewski and Dutton, 2001).

According to Wrzesniewski and Dutton’s (2001) original theoretical framework, job crafting can take three different forms: task, relational and cognitive crafting. Specifically, task crafting comprises altering the number, type and scope of tasks that employees need to fulfill at work. For instance, employees decide for themselves to take on or reduce the number of tasks, and introduce new means to complete those tasks to make their jobs easier or more interesting. Relational crafting involves exercising discretion to alter the quality and/or number of interactions with others encountered on the job. Examples include workers focusing on developing relationships with those who share similar interests, or distancing themselves from unpleasant colleagues. Cognitive crafting refers to the reframing of individuals’ perceptions and cognitive representations of their jobs. By changing the task, relational, and cognitive boundaries of their jobs, employees can shape the meaning of the job and, as a result, influence their work identities accordingly.

Alternatively, Tims and Bakker’s (2010) conceptualization of job crafting is based on the job demands-resources (JD-R) model. Although job crafting is initiated by the employees, it occurs within the context of their prescribed jobs, which are bounded by the specified tasks, expectations and structure imposed by the organizations. This definition emphasizes job characteristics that can be crafted across all occupations, namely job demands and resources. Job demands job characteristics that involve sustained physical or mental effort and are therefore associated with physiological and psychological costs. Although job demands could potentially evoke strain if they exceed employees’ adaptive capabilities (Bakker et al., 2007), they can also lead to positive outcomes if employees have enough resources to manage them. A challenging work demand may induce increased effort and satisfaction from the employee for achieving such a difficult task. Job resources are job characteristics that facilitate the achievement of work goals and promote personal growth (Bakker and Demerouti, 2007). By altering job demands and resources, employees can shape their jobs to match their individual abilities and desired goals. Tims et al. (2012) differentiated four dimensions of job crafting: (1) increasing structural job resources, such as task variety, opportunities for professional development, and job autonomy; (2) increasing social job resources, such as social support, supervisory coaching, and feedback; (3) increasing challenging job demands, which refer to taking on tasks that stimulate skills development or sense of accomplishment, such as new projects and higher levels of responsibility that are rewarding; and (4) decreasing the level of hindering emotional and cognitive job demands that interfere with the ability to achieve important work goals, such as role conflict and stressful interactions (Cavanaugh et al., 2000). Therefore, employees can craft their jobs to achieve better fit, helping them to maintain motivation and promote well-being.

### Older Workers and Job Crafting

Much of the empirical literature on job crafting thus far employs the conceptualization rooted in the JD-R model, proposing that employees focus on altering their job demands and resources in desirable directions (Bakker et al., 2012; Nielsen and Abildgaard, 2012; Kooij et al., 2016). Researchers have also suggested specific job crafting activities that are likely to be most relevant for older workers. Promotion-focused job crafting such as increasing structural and social resources motivates them to continue working beyond retirement age through the mediating mechanism of managing burnout (Lichtenthaler and Fischbach, 2016). Kooij et al. (2015), using the Selection, Optimization and Compensation (SOC) framework, identified three forms of job crafting for older workers: Accommodative crafting, which focuses on regulating losses, include delegating lower priority tasks, hiring an assistant, and reducing workload. Developmental crafting refers to strategies that focuses on growth, such as participating in workshops, using professional network for learning and partaking in professional organizations. Utilization crafting emphasizes employing workers’ existing skills, such as taking on tasks to activate previously unused skills and prioritizing attainable goals that are personally meaningful.

Job crafting was originally conceptualized as strategies that can be adopted across occupations and job ranks, yet behavioral changes to the tasks and relationships can be limited by job autonomy, task nature and organization structure (Tims and Bakker, 2010; van Wingerden and Niks, 2017). Cognitive crafting, which has received little research attention, can be a significant facet that would be especially valuable for older workers. We expand the definition of cognitive crafting beyond employees altering their view of the job, but also changing how they perceive their own role as workers. Instead of treating it as a mere coping mechanism, it can be another way for employees to proactively improve their work experience through aligning their actions and identities. Identity generally refers to “who the individual thinks he or she is and who is announced to the world in word and action” (Charon, 1992, p. 85). Specifically, work identity refers to how people perceive and define themselves at work (Wrzesniewski and Dutton, 2001), which affects the roles people take on and influences their subsequent behaviors and cognitions when performing the job. Work identities are important because they provide information about features that influence how people act, think and feel at work (Ashforth and Kreiner, 1999). As the formation of work identities is an active process, workers proactively create situations that confirm their favorable self-concepts (Schlenker, 1985). When there is a mismatch between workers’ sense of self and roles, workers may change their actions or modify their identities to fit work demands (Pratt et al., 2006). Cognitive crafting permits employees to ascribe additional meanings to the tasks that they do and cultivate their work identities, which in turn may motivate them to engage in specific crafting behaviors based on these meanings. Workers, thus, can reconcile the person-job misfit and maintain a positive sense of self. While it was not discussed in the conception by Wrzesniewski and Dutton, it is also possible for workers to cognitively disengage from their jobs in response to individual and environmental changes. Using the stereotype threat framework, utilizing cognitive crafting may help older workers to buffer age-related stereotype threat and protect their self-identity. Building on the life span theory of control, we propose that job crafting, particularly cognitive crafting, will be of increasing value as employees age.

### Value of Cognitive Crafting for Older Workers

The Life Span Theory of Control proposes that individuals throughout the life course need to balance their primary and secondary control strategies in order to maintain functional equilibrium (Heckhausen and Schulz, 1995). Primary control comprises actions directed at changing the ∖environment to fit individuals’ wants and needs, and secondary control aims at changing the individual self to be congruent with the environment. Researchers have argued for the functional primacy of primary over secondary control when it is attainable (Heckhausen and Schulz, 1995). Extending the Life Span Theory of Control to the context of job crafting, task and relational crafting are primary control strategies aimed at changing the job itself by changing what individuals do and who they interact with at work. On the other hand, cognitive crafting is a secondary control strategy, enabling employees to achieve changes in their own perceptions of the job. Yet, primary control strategies may not always be feasible. Although the motivation to strive for primary control remains stable, people’s capabilities to do so may decline with age due to both within-person and environmental constraints. When this occurs, secondary control plays an increasingly important role. For instance, older workers are likely to face a certain extent of decline in physical capabilities which they have little control. While they may be able to apply primary control strategies in other aspects such as seeking out tasks that rely on cognitive abilities, the focus of resources may shift to regulate their thinking and emotions as this is an uncontrollable situation. Secondary control not only helps people to cope with losses of primary control, but it facilitates primary control through contributing to the selection of goals pursued (Heckhausen and Schulz, 1995). By altering how one thinks of primary control failures, secondary control buffers the impact on self-concept and preserve motivational resources for utilizing the primary control approach again in the future. Similarly, when certain forms of task and relational crafting are not attainable, cognitive crafting can be one particularly valuable way for older workers to cope and redirect resources to other areas that are personally important.

While there are few empirical studies on cognitive crafting, research on highly stigmatized occupations sheds light on techniques that workers might use to create more meaning in their jobs (Ashforth and Kreiner, 1999). For example, reframing involves reconstructing the job in a way that differs from its apparent meaning, tying in more abstract value with greater purposes. As older workers tend to have longer tenure, they may perceive that their work not only contributes to the everyday operation of the organization, but as being instrumental to the organization’s growth over the years. This, in turn, would allow them to maintain and further develop positive worker identities. Since ageism is one of the most prevalent discrimination at workplaces, older employees are constantly faced with negative age stereotyping cues (Lamont et al., 2015). Individuals may shift to alternative means to meet their overarching needs for a positive sense of self in response, exemplified by findings on employing self-affirmation to combat stereotype threat (Martens et al., 2006; Sherman et al., 2013). Instead of tending to the negative stereotypes, older workers, due to their predisposition to focus on the positive, may be better able to redirect their limited resources toward cognitive crafting and recognize age as a badge of honor that symbolizes their accumulated experience and wisdom. This allows them to preserve a positive sense of self as workers, which is vital for their self-esteem and job performance. It has been theorized that older individuals are threatened the most by self-concept based stereotype – the possibility that negative age-related stereotypes are personally true of themselves (Barber, 2017). Cognitive crafting may allow older workers to refine how they perceive themselves and to view age in a more positive light. This might offset the negative effect of stereotype threats on self-concept and performance, and maintain positive overall work identities.

Cognitive crafting may be particularly essential in situations where person-job fit is the result of situations that older workers have little control. When changing tasks and relationships at the job are not possible, older workers may engage in identity patching, that is changing their sense of self to make sense of workplace situations (Pratt et al., 2006). Through cognitive crafting, older workers may choose to place less value on certain aspects or their jobs as a whole and move toward the career disengagement stage as suggested by Super’s Career Stages theory (Super, 1980). For instance, if their needs for emotional involvement have been satisfied outside of work, such as being caregivers for their families or pursuing hobbies, older workers may place less value in cultivating positive relationships at work. Or, when employees are chronically faced with age stereotyping at the workplace, cognitively disengaging from work itself allows them to persist without doing further damage to their self-identity (Woodcock et al., 2012). Although it can be costly to the organizations, cognitive crafting through disengaging and devaluing aspects of work can potentially improve older employees’ work experience.

As employees age, cognitive crafting may become increasingly important as a means for both maintenance and development of task and relational crafting. Life Span Theory of Control proposes that secondary control optimizes the selectivity of primary control targets. This may be accomplished by promoting the value of selected goals while disengaging from those that are no longer attainable, and enhancing individual’s self-confidence (Heckhausen and Schulz, 1995). Due to decreases in job mobility, it is difficult for older workers to change jobs. However, they can shift their attention to job features that are extrinsically or intrinsically rewarding for them personally (Ashforth and Kreiner, 1999). Because of their longer time in the workforce, older workers tend to have a better understanding about their preference in tasks, as well as their strengths and weaknesses (Helson et al., 1995). Therefore, they are more capable of optimizing their resources and directing them to suitable crafting practices. For instance, older workers may choose to learn how to navigate new computer systems based on their interest in technology, focusing their attention on achieving a sense of mastery despite common stereotypes. Or, they may place the value of their jobs on building meaningful social relationships with colleagues as their purpose, instead of putting in extra effort for a promotion. It also has been suggested that older workers tend to engage in job crafting using their personal strengths placing more value on serving their organizations than job crafting based on their own personal interests (Kooij et al., 2017). Through refocusing, older workers can appreciate job features that make the job worthy of their time and energy. These job features then serve as targets for them to employ in further primary crafting, that is, task and relational crafting, so that their behaviors are more consistent with their identities.

### Value of Task and Relational Crafting for Older Workers

Through employing task and relational crafting, older workers can directly manipulate their daily job duties and interactions with other employees as suggested by previous researchers (Kooij et al., 2015). As older employees often have held the same job for a long time, tasks that once were challenging and meaningful can become routine and lackluster (Robson and Hansson, 2007; Hornung et al., 2010). They are often given more routinized job assignments due to stereotypes rooted in ageism (Salthouse and Maurer, 1996). Thus, older workers may no longer find their jobs to be satisfying their needs for mastery and achievement. Task crafting allows older workers to proactively seek out opportunities that interest them and change how they perform their jobs to fit their values. For example, older individuals who have a passion for learning may actively look for training and volunteer to join challenging projects, even if their organizations may not consider them to be the best candidates for these openings. Furthermore, they can alter, refine or minimize interactions with others at work through relational crafting to match their needs and wants. For instance, generativity motives, which is the tendency to focus on helping others, the society as a whole and future generations instead of self, increase with age (McAdams et al., 1993). Kanfer and Ackerman (2004) argued that employees who are motivated by such motives would pay attention to the collaborative process of goal accomplishments, rather than outcomes, on the job. As a result, the desire to pass on knowledge to their younger colleagues may become the primary driving force for some older workers, thus changing how they approach their work. Instead of aspiring to advance their own careers, older workers may prefer to take on mentoring roles to train others on areas of their strengths. At the same time, due to the increasingly more limited future time perspective with age, older workers may become more selective about who they invest their time in. They may distance themselves from colleagues and supervisors who do not share similar core values, which helps improve the alignment between their actions at work and who they perceive themselves to be as workers. These crafting strategies are also consistent with the SOC theory. Primary control crafting behavior provides an avenue for older workers to direct their limited resources to job responsibilities and relationships that are personally motivating and essential features to their identities, which consequently improve the alignment between their jobs and themselves.

## Conclusion

Aging at work is a dynamic process. Changes in employees’ motives and competencies, along with job changes occurring over their work histories, increase the likelihood that older workers will experience person-job misfit. Through using various strategies of job crafting, older employees can realign their person-job fit and maintain congruent work identities. As job crafting is initiated by employees themselves, it would be able to accommodate the substantial individual differences existing among older workers. While most of the empirical literature, thus far, focuses on job crafting through balancing job demands and resources, we believe that it will be of theoretical value to revisit the conceptualization proposed by Wrzesniewski and Dutton (2001) to examine cognitive crafting. As a secondary control strategy, it serves as an additional means for workers to cultivate meaningful work identities and give priority to job features that are personally important for additional primary job crafting. More research will further demonstrate the potential value of job crafting for this unique population.

## Author Contributions

CW played a primary role in the conception and drafting of the manuscript. LT played a major role in drafting of the manuscript and providing critical revision to the manuscript.

## Conflict of Interest Statement

The authors declare that the research was conducted in the absence of any commercial or financial relationships that could be construed as a potential conflict of interest.
